# Transforming transnasal endoscopy services: A multicentre service evaluation pilot project

**DOI:** 10.1016/j.clinme.2025.100300

**Published:** 2025-03-04

**Authors:** Mohamed Hussein, Jason Dunn, Farhana Sultana-Miah, Sami Hoque, Ahmed Albusoda, Esra Asilmaz, Laura Marelli, Regina Raymond, Mohsen Eldragini, Michael Grimes, Shraddha Gulati, Juriese Saramosing, Mayur Kumar, Eleanor Knights, Vinay Sehgal, Paul Maxwell, Arun Rajendran, Shamima Padaruth, Sophie Stevens, Sergio Coda, Edward Despott, Saswata Banerjee

**Affiliations:** a Guy's and St Thomas’ NHS Foundation Trust; b NHS England; c Barts Health NHS Trust; d Homerton University Hospital NHS Foundation Trust; e Barking, Havering and Redbridge NHS Foundation Trust; f Royal Free London NHS Foundation Trust; g Kingston Hospital NHS Foundation Trust; h Kings College Hospital NHS Foundation Trust; i University College London NHS Foundation Trust; j North Middlesex University Hospital NHS Foundation Trust; k The Hillingdon Hospitals NHS Foundation Trust; l Imperial College Healthcare NHS Foundation Trust

**Keywords:** Transnasal endoscopy, UGI Cancer, Endoscopy

## Abstract

**Objectives:**

To assess the impact of pilot transnasal endoscopy (TNE) services on workforce efficiency, allocated procedure times and patient tolerance of procedures. The aim was to also understand the challenges of setting up a TNE service.

**Methods:**

Six-month data were collected from ten sites. Data captured included productivity, performance, workforce numbers, facilities and quality metrics.

A patient survey was done to capture patients’ experience. An eight Likert-style and open question survey was designed and used. Pilot sites were visited using a semi-structured interview process.

**Results:**

About 30% of the pilot sites carried out the TNE service outside of the endoscopy unit. There is an overall 25% improvement in workforce efficiency with TNE.

Of those patients who had both a TNE and an oesophagogastroduodenoscopy, 78% reported that having the TNE procedure was a better experience.

All sites reported that they will continue providing TNE beyond the pilot period. Sites carrying out TNE reported a high satisfaction with the services. Overall satisfaction with the quality of TNE imaging was very high.

**Conclusions:**

This multicentre pilot project shows evidence that the integration of TNE services has a positive impact in increasing capacity and patient satisfaction. This should set the scene for scaling this up on a wider capacity. TNE services, particularly with an introduction into outpatients, will improve service capacity in endoscopy, patients will tolerate the procedures more, national 2-week wait and Faster Diagnosis Standard targets will improve, and it is potentially more cost efficient overall.

## Highlights

• Transnasal endoscopy (TNE) presents an alternative to a standard per oral gastroscopy.
• TNE can be done in outpatients and therefore improve endoscopy capacity.
• TNE services improve workforce efficiency.
• TNE is better tolerated than a standard per oral gastroscopy by the majority of patients.

**Summary box**

**What is already known on the topic?**

There is generally a low uptake of transnasal endoscopy (TNE). It is better tolerated than a standard gastroscopy, can be done with local anaesthetic and offers high-quality imaging. It presents an attractive alternative to a per oral gastroscopy that can be performed outside the endoscopy unit and improve earlier detection of upper gastrointestinal cancers.

**What was found?**

This multicentre pilot project shows that the integration of TNE services has a positive impact in increasing capacity, workforce efficiency and patient satisfaction. It provides a model that can be scaled up nationally.

**How might this study affect practice?**

It provides a model that can be scaled up to improve faster diagnosis rates and maximise capacity for 2-week wait endoscopy to rule out or diagnose cancer. Further space can be created in the endoscopy unit for colonoscopy and therapeutic procedures to reduce backlogs.

## Introduction

Conventional oesophagogastroduodenoscopy (c-OGD) is the gold standard for the investigation of pathology of the upper gastrointestinal (GI) tract. It makes up approximately half of all GI procedures in the UK. The COVID-19 pandemic has had a significant effect on endoscopy services nationally. A national analysis of endoscopy data showed that there was a significant backlog.^1^ There is a significant need to improve capacity in endoscopy in the UK to meet targets.^2^ The 2021 JAG census showed that 57.9% of NHS services met urgent cancer waits, 13.4% met surveillance waits and 17.9% met routine waits.^3^

Transnasal endoscopy (TNE) is performed with the use of ultrathin endoscopes via the nasal route. This bypasses the oral cavity, which is an area of high sensitivity that can trigger a gag reflex. This means better procedure tolerance and patient satisfaction.^4^ It is performed normally without sedation and therefore more convenient. There is an increasing use of TNE in the UK; however, it is only available in a few centres. Case studies have shown that TNE can be easily adopted by endoscopists experienced in c-OGD.^4^^,^^5^

A systematic review showed that the technical success rates of non-sedated TNE was equivalent to that of c-OGD; however, TNE had a higher patient tolerability and acceptability and provoked less cardiovascular stress.^6^ Advances in endoscopic technology means that more modern TNE endoscopes provide high-quality imaging and functionality characteristics, which are similar to gastroscopes.^5^ It is useful for the assessment of patients with advanced oesophageal strictures and also for the insertion of nasojejunal tubes.^7^

There is a move towards green endoscopy with sustainable practices and waste reduction. TNE would help support reducing the environmental impact of endoscopy, with minimal use of single-use plastics required for a c-OGD such as mouthguards, needles and syringes.^2^

The high patient satisfaction, low complication rate and potential cost savings have made TNE an attractive alternative to c-OGD for screening of upper GI pathology.^8^ The better tolerability would mean improved completion rates of procedures. TNE provides an opportunity to perform endoscopy in outpatients or other alternatives spaces outside the endoscopy unit, therefore creating capacity in endoscopy for therapeutic procedures. The advantages that it has over potential alternatives like capsule endoscopy are that a biopsy can be taken at the time of the procedure if any pathology is identified.

Despite all the advantages offered by TNE, it is underutilised in the UK. In 2019, 26,685 TNE procedures were performed (less than 3% of all OGDs in the UK).^2^^,^^9^ There is a need to scale up the use of TNE to meet the increasing demand in endoscopy.

## Aims

The aims of this project are:

- to assess the impact of TNE services on the overall workforce capacity and efficiency
- to assess tolerance and patient satisfaction of TNE
- to assess the impact of TNE on the overall allocated procedure time
- to understand the challenges of setting up a TNE service.

## Methods

### Site recruitment and funding

**Fig. 1:**
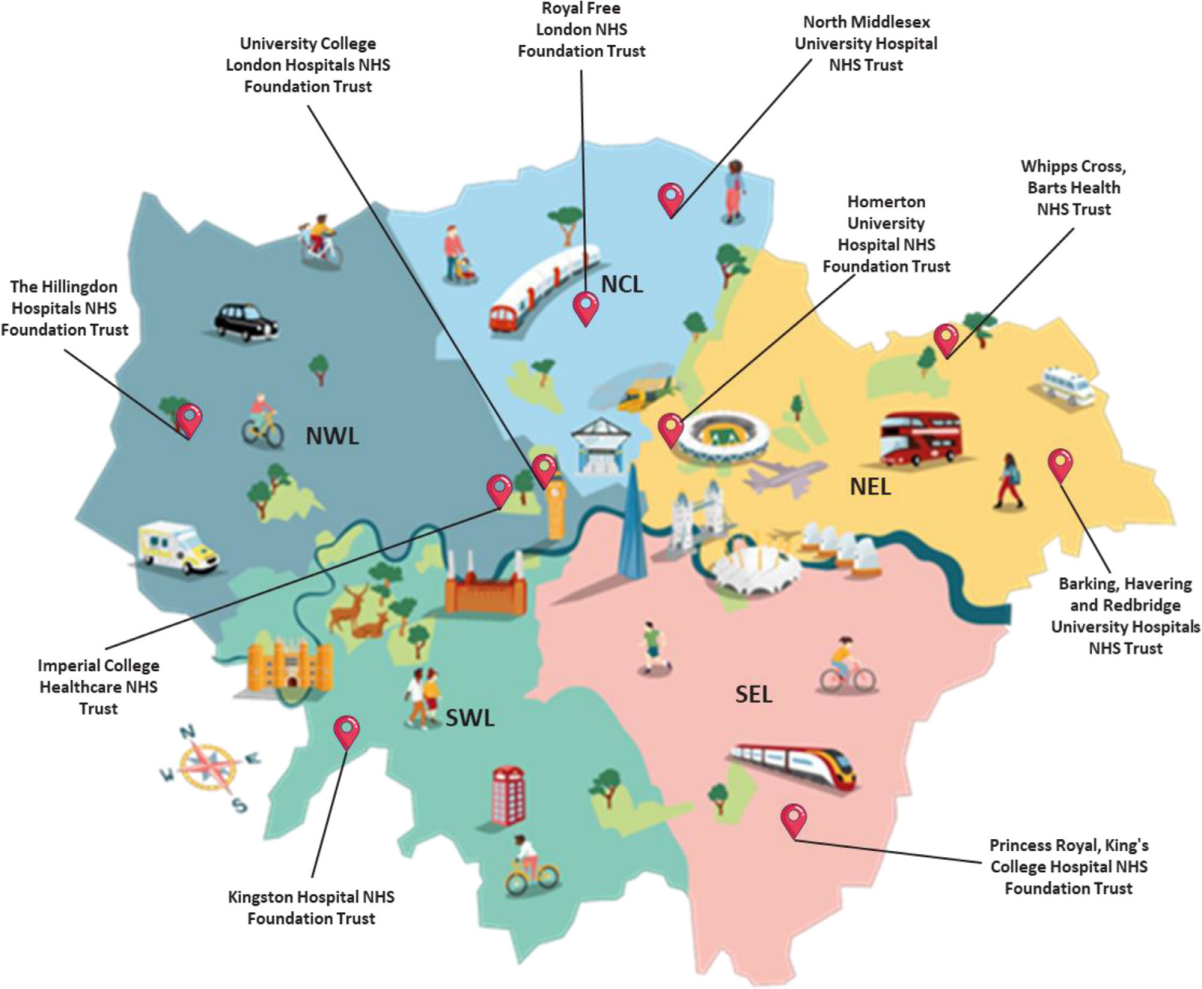
The 10 London sites recruited into the 6-month pilot TNE project.

Ten pilot sites in London were recruited to provide operational insights into integrating TNE in the outpatient services over a 6-month period (October 2022 – March 2023) (Fig. 1). The pilot was funded with £2.48 million capital funding and a further £150,000 revenue funding.

We conducted a retrospective service evaluation of the pilot project as part of a standard of care practice. No identifiable patient information was shared and there was no impact on patient care, therefore, institutional review board (IRB) approval was not necessary. The evaluation followed a decision-making process approved by the London Diagnostic Programme Board, which includes representatives from all integrated care boards (ICBs). Additionally, all trusts submitted their capital and revenue bids, which were reviewed and approved by the national team. A memorandum of understanding (MOU) was established between the trust and NHS England (NHSE), outlining contributions and the regular reporting of the implementation progress to NHSE regional teams, ensuring that the benefits of the scheme were delivered in line with the agreement.

### Facilities

TNE could be done on the endoscopy unit, community diagnostic centres, mobile units or in outpatients. The decision on where to perform TNE was reached on a site-by-site basis depending on space availability. Three brands of endoscopes were used – Olympus, Fuji and Pentax.

Upper GI referrals from GPs at each site were triaged by clinical staff and selected for TNE if appropriate, depending on local criteria including 2-week wait referrals. Pre-procedure processes including the written information was sent by post to the patients in all sites.

### Evaluation methods

Six-month data were collected. Data captured included productivity and performance, workforce numbers, facilities and quality metrics.

Sites used a uniform set of metrics for evaluating performance. Data were gathered from the site-specific recording systems.

### Patient survey

A patient survey was used to capture patients’ experience using an eight Likert-style and open question survey. This was done in either paper or electronic format.

### Site visits

Sites were visited using a semi-structured interview process. The outcome measures assessed during the interview included assessment of technology and equipment, technical competence of staff, leadership, relationship with other services, coordination of work, patient experience and overall quality of patient care.

### Statistical analysis

Descriptive statistics were used to present the categorical data, which were reported as absolute numbers with proportions and percentages. Continuous variables were reported as medians with interquartile ranges. Kolmogorov–Smirnov statistics were used to check the distribution of continuous variables. The independent *t*-test was used for comparison between groups. A *P* value less than 0.05 was considered statistically significant.

## Results

### Performance outcomes

There were 26,755 upper GI referrals during the 6-month pilot period. About 2,429 TNEs were booked. About 24,326 patients were booked for a standard OGD.

On average, sites ran two TNE lists per week and a median of 8 (IQR 6-8) patients per list.

The did not attend (DNA) rate for the TNE procedures was 10% (241/2,429). The DNA rate for c-OGDs in the same period was 5% (1,272/24,326). The London average DNA rate for outpatients in April 2023 was 10.8%.

50% (five out of 10) of the pilot trusts allocated 15 min per TNE procedure. 30% (three out of 10) of the trusts allocated 20 min for a TNE procedure. Two out of the 10 hospitals allocated 30 min per TNE procedure.

The average allocated procedure time for a c-OGD was 16 min 30 seconds, versus the average allocated procedure time for a TNE of 19 min and 30 seconds (*P* = 0.09). The end-to-end pathway for TNE is quicker than a standard OGD as no recovery time is required.

Most pilot sites had captured TNE procedures as a day case. Two sites had recorded the TNE procedure as an outpatient procedure.

About 13% of patients were found to not be suitable for a TNE procedure across four sites. The conversion rate from TNE to a c-OGD on the day of the procedure was 7%. The conversion rate to a future c-OGD was 6%.

There was a 54% biopsy rate across eight pilot sites.

### Facilities and workforce metrics

30% (three out of 10) of the pilot sites carried out the TNE service outside of the endoscopy unit.

On average, a workforce total of three people including nurses and the endoscopist were required per TNE procedure. For c-OGD across all 10 sites, a mean of four members of staff were required per procedure. There is an overall 25% workforce efficiency with TNE**.**

**Fig. 2:**
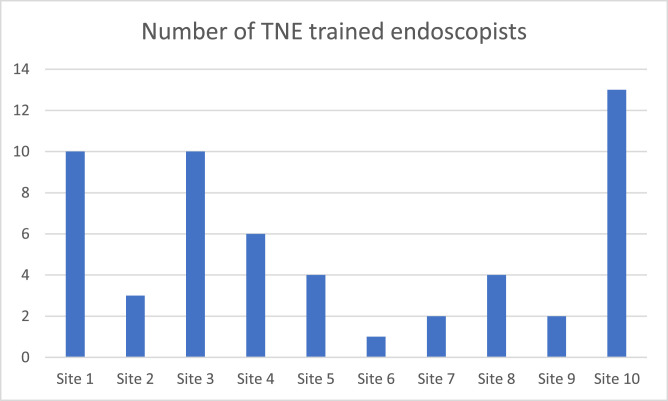
Number of endoscopists trained to do TNE at each of the pilot sites.

About 55 endoscopists were trained across all 10 sites to perform TNE (Fig. 2). There was an average of two TNE lists per week across all 10 pilot sites.

In 50% (five out of 10) of pilot sites, the TNE lists were run by nurse endoscopists.

### Patient survey

A total of 112 patients took part from six sites. The four remaining sites did not take part or used their own version of a questionnaire.

**Fig. 3:**
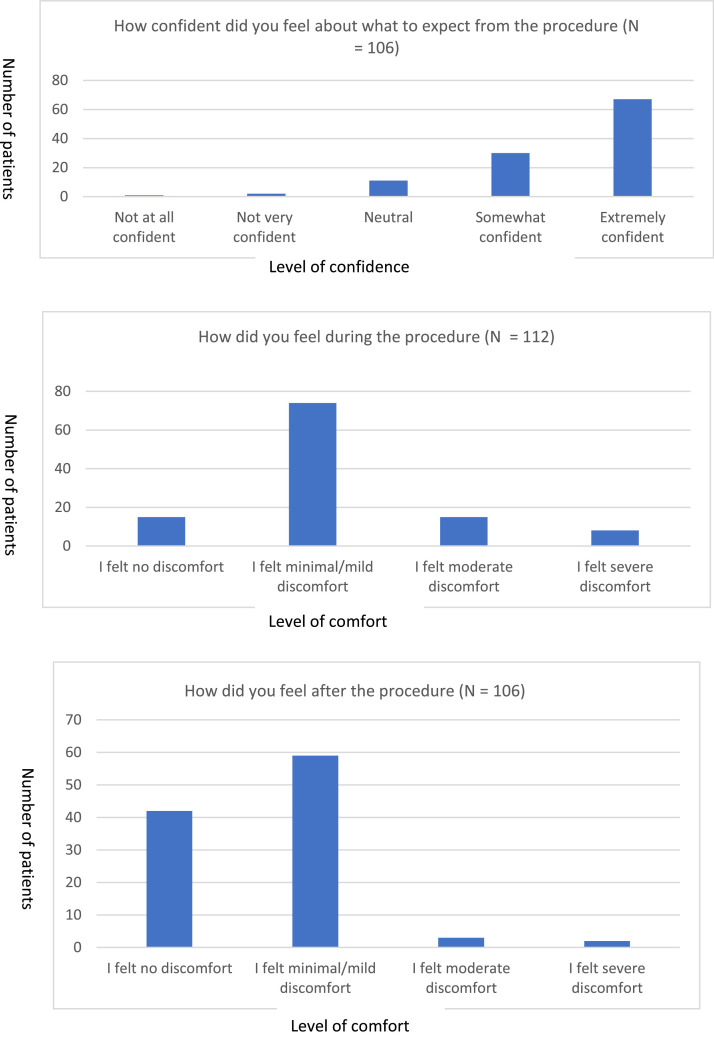
Patient survey outcomes about the TNE procedure experience.

About 87% (97 out of 111) of patients felt either ‘extremely confident’ or ‘somewhat confident’ in what to expect from the TNE procedure. About 98% of patients felt that they had understood the benefits of the procedure. About 97% of patients felt they clearly understood the potential risks (Fig. 3).

About 95% (101 out of 106) of patients felt no/minimal/mild discomfort after the procedure.

When asked about a comparison between a TNE and a c-OGD procedure, 96 patients responded. Of those that had had both (46 patients), 78% (36 patients) reported that having the TNE procedure was a better experience.

### Site visit outcomes

All sites reported that they will continue providing TNE beyond the pilot period. Sites carrying out TNE reported a high satisfaction with the services. Overall satisfaction with the quality of TNE imaging was very high.

Some sites reported a lack of awareness from primary care and some confusion that TNE may be exclusively an ENT (ear, nose and throat) procedure.

Lack of space was the most common reason for not being able to run TNE in outpatients. One benefit cited for running it in the endoscopy suite was direct access to gastroenterologists in case of an emergency or opinion. Some sites reported the use of the expertise of the ENT department in the set-up phase. Most sites reported an ambition to perform TNE in outpatients in future in order to create additional capacity.

One of the benefits cited with the regards to the pilot was the upskilling of staff to perform a TNE procedure. In most cases, patients were discharged immediately post-procedure with no recovery time.

## Discussion

International uptake of TNE has been low. A study showed that 34% of clinics and 9% of hospitals used TNE as first line in Japan versus 1% worldwide.^2^ There were similarly low numbers in the 2019 JAG census. This may be due to lack of formal training programmes and accreditation for TNE. Other pilot studies in the UK have demonstrated that TNE is easy to learn, despite varying levels of endoscopic experience, with a quick learning curve.

Our multicentre pilot evaluation experience shows the integration of TNE services has a positive impact in increasing capacity, workforce efficiency and patient satisfaction.

In our pilot, 78% of patients reported a better experience with TNE compared to a standard OGD. About 95% of patients who had a TNE reported no or minimal discomfort during the procedure. This shows that it is a well-tolerated procedure and would minimise the need for general anaesthetic procedures, of which there is limited capacity. This supports evidence from other studies which show that TNE is better tolerated than a c-OGD.^2^ There can be discomfort in the nasal area, which can be rectified with adequate topical nasal spray. A prospective randomised trial compared c-OGD versus TNE where the majority of the TNE patients (85%) found the procedure better than expected versus 54.5% in the c-OGD group (*P* < 0.001).^10^

In our pilot, 87% of patients felt confident of what to expect from a TNE procedure and 98% felt that they understood the benefits of the procedure. Explanation of the procedure and reassurance to patients during booking and in the primary care setting will help reduce DNA rates. This will help maximise use of list capacity and will minimise conversion rates from TNE to a sedated c-OGD by minimising any anxiety on the day of the procedure. In the pilot, there was a 10% DNA rate with TNEs. This can be improved with further education of GPs to introduce the concept of TNE in primary care to manage patient expectations with having the procedure. A prospective multicentre study by Sami *et al*, where patients had both TNE and c-OGD on the same day, showed that a higher proportion of patients preferred a TNE (54.2%) versus a c-OGD (16.7%) (*P* < 0.001).^11^

The pilot project showed that the TNE services helped improve workforce capacity. The TNE procedure required 25% less staff than a c-OGD due to no sedation and no recovery time. About 50% of the procedures were performed by nurse endoscopists, therefore freeing up consultants for other clinical activity. This allows for more flexibility in the workforce and minimising list cancellations. This is supported by results from a pilot study where the TNE procedures were supported by only one nurse (band 5 level) and one healthcare assistant.^2^

An important factor in allowing flexibility in the workforce is endoscopists being trained to perform TNE. In our pilot, 55 endoscopists were trained to perform TNE across 10 hospitals. This will need to be replicated nationally and there is an argument now to include TNE as part of mandatory JAG accreditation, including attendance on a TNE course. This will allow for flexibility in expansion of TNE services in departments.

About 30% of the pilot sites carried out the TNE service in the outpatient setting. This is a positive sign of progression in the right direction. This will allow for more capacity for therapeutics in endoscopy and also for one-stop clinics in outpatients. During the site visits, all sites showed an ambition to transition the TNE services to outpatients in future; however, the biggest challenge was the lack of space. This could be the same challenge nationally, which would need to be overcome.

It has been found that the diagnostic yields of upper GI pathology is similar with TNE.^12^ A pilot TNE service study by Lim *et al* showed an inlet patch detection rate of 5.7% with TNE which is comparable with that of c-OGD.^2^ Despite the smaller 2.4 mm working channel in a TNE, studies have shown that biopsies taken meet criteria required to reach a diagnosis. Therefore, TNE would potentially have an important role in surveillance procedures such as eosinophilic oesophagitis, where biopsies are routinely required. These patient cohorts would need repeated endoscopies and, as previous studies have shown, with greater tolerability and satisfaction with TNE these patients are then more likely to undergo repeat procedures in future.^12^ Walter *et al* prospectively compared biopsies obtained in c-OGD versus TNE. There was no significant difference in the rate of definitive histological diagnosis from biopsies.^13^ A more standardised approach is needed nationally in terms of the pre-procedure preparation, such as timing of nasal spray pre-TNE in order to optimise tolerance of the procedure for patients.

With advances in endoscopic technology, TNE endoscopes now have a high image quality.^14^ Advances in the ultrathin endoscopes have allowed the gap in differences between a TNE scope and standard OGD endoscope to be bridged.^15^ A study evaluated the use of TNE in the assessment for gastric cancer and found that narrow-band imaging assessment using the ultrathin TNE was effective for improving endoscopic diagnosis.^16^

The biopsy rate in our pilot was 54%, which was in keeping with previously reported rates during standard gastroscopy. One study showed that out of 8,572 patients who had undergone a gastroscopy, 57% of patients underwent a duodenal biopsy.^17^ It can be extrapolated from this that the quality of imaging during TNE is of high enough quality for a good optical diagnosis and that overuse of biopsies does not occur. This avoids any extra costs.

The mean time allocated across the sites to TNE was slightly longer than a standard gastroscopy. A likely reason for this was that, in one centre, 30 min was allocated to include both a clinic appointment and an endoscopy procedure. By default, the end-to-end journey time for patients is less with a TNE than a standard OGD due to no requirement for sedation and no recovery time. This will allow for a greater number of procedures to be done on the list compared to a c-OGD. The JAG-mandated criteria for a list are 12 points. This would potentially allow for an argument to increase the points on a TNE list, therefore increasing capacity and potentially increasing financial revenue to trusts.

There was a conversion rate of 13% from TNE to OGD. The main reasons for this were the poor tolerance of the TNE and patients requesting sedation on arrival. After reassurance, most patients who initially preferred sedation went on to proceed with TNE. The main ways to try and overcome this issue is through education of GPs about TNE. This will help manage patient expectations, minimise anxiety and maximise TNE completion rates. It would also be important to induct the administration team with regards to how to explain TNE to patients to maximise patient reassurance during procedure booking. Another factor maybe a need for more structured TNE training and certification with JAG key performance indicators being met, just like for c-OGD. This will ensure that endoscopists are confident at the point of independence with passing of the endoscope through the nasal passage with minimal patient discomfort.

One of the strengths of this pilot evaluation project is that it provides real-world data from different centres and gives a clear understanding of what challenges need to be overcome in order to scale this up further. One of the key challenges to overcome is the ability to do TNE in outpatients as part of a one-stop service. There will be some starting investment costs for services set-up outside of the endoscopy unit, for example ensuring there is a sufficient number of endoscopes. These costs will be far outweighed with the longer-term gains of maximising capacity, reducing endoscopy waiting lists and meeting cancer targets.^2^

Limitations of the pilot study are the small number of patients. This was due to inclusion of a limited number of sites over a limited period of 6 months during this pilot phase. The lack of JAG criteria means there is a lack of standardisation in uptake and will be particularly important when rolling out nationally. Another limitation was the end-to-end journey time from admission to discharge for TNE and c-OGD was not recorded. This would be an important metric to include in future projects. This will likely confirm that this time is shorter for a TNE due to the lack of sedation. This would potentially improve list efficiency and increase capacity. Another limitation was the reason for every conversion from a TNE to c-OGD was not recorded to give the percentages of each cause. The overall causes were recorded as part of a structured interview at each centre. A further limitation is that centres were allowed to follow local protocol. Ideally in future projects, we would aim for a standardised protocol, for example for timing of administration of nasal spray or use of simethicone drinks pre-procedure. This is something that can be standardised as part of national TNE service guidelines.

From the lessons learnt, in particular from the site visits, there are clear recommendations that should be potentially made to provide the most achievable and sustainable framework for replication of an effective TNE service. Trusts would need to make efforts to try and increase capacity within the endoscopy unit or in any appropriate space outside the endoscopy rooms. Consideration should be made into embedding the TNE pathway as part of the GP direct access implementation, which would allow for a more effective referral pathway for TNE. More encouragement is needed to train more nurse endoscopists and GI trainees to perform TNE, which would allow for upskilling, career progression and increased delivery capacity. Patient and primary care educational videos and leaflets would help raise awareness of the TNE pathway.

All pilot sites have agreed to run the TNE service as part of the business-as-usual service. Many more trusts have shown interest in setting up similar services at their sites.

## Conclusions

This multicentre pilot shows evidence that the integration of TNE services has a positive impact in increasing capacity and patient satisfaction. Barriers have been identified for the introduction of this service, with potential solutions. This should set the scene for scaling this up on a wider capacity, particularly outpatients. This will improve service capacity in endoscopy, national 2-week wait and Faster Diagnosis Standard targets will improve and there will be an improvement in workforce efficiency.

## Ethical approval and consent to participate

We conducted a retrospective service evaluation of the pilot project as part of a standard of care practice. No identifiable patient information was shared and there was no impact on patient care, therefore, IRB approval was not necessary. The evaluation followed a decision-making process approved by the London Diagnostic Programme Board, which includes representatives from all integrated care boards (ICBs). Additionally, all trusts submitted their capital and revenue bids that was reviewed and approved by the national team. A memorandum of understanding (MOU) was established between the trust and NHS England, outlining contributions and the regular reporting of the implementation progress to NHSE regional teams, ensuring that the benefits of the scheme were delivered in line with the agreement.

Patients consented to have the transnasal endoscopy procedure as part of standard of care procedure. Consent was not required for inclusion of data as they was all anonymised and a service evaluation of standard of care services.

## Funding

The pilot was supported by funding from NHS England's Digital Diagnostics Programme, under the PDC scheme: CENT – LIMSD – Digital Diagnostic Programme 21–22 and by Health Education England (HEE), now part of NHS England for the fiscal year 2021–2022. The funding sources had no direct involvement in the pilot study itself.

## Data availability statement

The data that support the findings are available from the corresponding author upon reasonable request.

## CRediT authorship contribution statement

**Mohamed Hussein:** Writing – review & editing, Writing – original draft, Formal analysis. **Jason Dunn:** Writing – review & editing, Methodology, Conceptualization. **Farhana Sultana-Miah:** Writing – review & editing, Project administration, Formal analysis, Data curation, Conceptualization. **Sami Hoque:** Writing – review & editing, Data curation, Conceptualization. **Ahmed Albusoda:** Writing – review & editing, Data curation. **Esra Asilmaz:** Writing – review & editing, Data curation. **Laura Marelli:** Writing – review & editing, Data curation. **Regina Raymond:** Writing – review & editing, Data curation. **Mohsen Eldragini:** Writing – review & editing, Data curation. **Michael Grimes:** Writing – review & editing, Data curation. **Shraddha Gulati:** Writing – review & editing, Data curation. **Juriese Saramosing:** Writing – review & editing, Data curation. **Mayur Kumar:** Writing – review & editing, Data curation. **Eleanor Knights:** Writing – review & editing, Data curation. **Vinay Sehgal:** Writing – review & editing, Data curation. **Paul Maxwell:** Writing – review & editing, Data curation. **Arun Rajendran:** Writing – review & editing, Data curation. **Shamima Padaruth:** Writing – original draft, Data curation. **Sophie Stevens:** Writing – review & editing, Data curation. **Sergio Coda:** Writing – review & editing, Data curation, Conceptualization. **Edward Despott:** Writing – review & editing, Data curation, Conceptualization. **Saswata Banerjee:** Writing – review & editing, Methodology, Funding acquisition, Data curation, Conceptualization.

## Declaration of competing interest

The authors declare the following financial interests/personal relationships which may be considered as potential competing interests:

The pilot project was funded with capital funding to allow set up of the services across the ten sites (Written in Methods section) reports financial support was provided by NHS England. If there are other authors, they declare that they have no known competing financial interests or personal relationships that could have appeared to influence the work reported in this paper.
